# Neutrophil diversity in inflammation and cancer

**DOI:** 10.3389/fimmu.2023.1180810

**Published:** 2023-04-26

**Authors:** Silvia Carnevale, Irene Di Ceglie, Giovanna Grieco, Anna Rigatelli, Eduardo Bonavita, Sebastien Jaillon

**Affiliations:** ^1^ IRCCS Humanitas Research Hospital, Milan, Italy; ^2^ Department of Biomedical Sciences, Humanitas University, Milan, Italy

**Keywords:** neutrophil, inflammation, cancer, innate immunity, tumor immunology

## Abstract

Neutrophils are the most abundant circulating leukocytes in humans and the first immune cells recruited at the site of inflammation. Classically perceived as short-lived effector cells with limited plasticity and diversity, neutrophils are now recognized as highly heterogenous immune cells, which can adapt to various environmental cues. In addition to playing a central role in the host defence, neutrophils are involved in pathological contexts such as inflammatory diseases and cancer. The prevalence of neutrophils in these conditions is usually associated with detrimental inflammatory responses and poor clinical outcomes. However, a beneficial role for neutrophils is emerging in several pathological contexts, including in cancer. Here we will review the current knowledge of neutrophil biology and heterogeneity in steady state and during inflammation, with a focus on the opposing roles of neutrophils in different pathological contexts.

## Introduction

Neutrophils represent the largest population of circulating leukocytes in humans and play a central role in acute phase responses (1). Given their inability to proliferate and their short lifespan, neutrophils are considered poorly adaptive cells with limited effector functions (2). This hypothesis has been challenged by recent discoveries made possible by new technologies. For instance, the lifespan and heterogeneity of neutrophils have been re-evaluated in the last years (3–8).

The reassessment of neutrophil biology revealed their plasticity under healthy conditions or during inflammatory processes and diseases, such as in cancer (9–11). Neutrophils are well known for their protective role in response against invading pathogens (12). However, they can sustain harmful and pathological inflammation in some conditions such as immune-mediated diseases, fibrosis, and cancer (13–15). In these pathologies, the presence of neutrophils or molecules associated with their recruitment have frequently been used as markers of severity and associated with poor clinical outcomes (9, 14–26). In contrast, recent findings have challenged this view and suggested that a better understanding of neutrophil plasticity and heterogeneity was needed to explain their different roles in pathological conditions (13, 23, 27). Here, we will discuss the current knowledge of neutrophil biology and diversity in steady state and in immune-related diseases, including in cancer.

## Neutrophil development, recruitment, and effector mechanisms

### Neutrophil development in steady state and inflammation

The half-life of neutrophils in circulation ranges from 6 to 8 hours. Therefore, their presence in blood requires a constant replenishment from the bone marrow (BM) (1, 28). The process of granulopoiesis is tightly regulated by the signalling of granulocyte-colony stimulating factor (G-CSF) and granulocyte–macrophage colony-stimulating factor (GM-CSF) (29). Accordingly, G-CSF and GM-CSF-deficient mice displayed severe and chronic neutropenia (30, 31).

In the bone marrow (BM), hematopoietic stem cells (HSCs) give rise to either common myeloid progenitors (CMP) or common lymphoid progenitors (CLP) (28). The CMP differentiate into granulocyte monocyte progenitors (GMP) or megakaryocyte erythroid progenitors (MEP), while CLP differentiate into T lymphocytes, B lymphocytes or NK cells (29). Then, the GMP differentiate into more mature cells referred to as myeloblasts, which subsequently differentiate into promyelocytes, myelocytes, metamyelocytes, and band neutrophils (29).

Granulopoiesis is defined by a specific and highly regulated transcriptional program where several transcription factors are involved, including CCAAT/enhancer binding proteins (C/EBPs), GATA-1 and PU.1 (28, 32, 33). C/EBPs comprise a family of six transcription factors (C/EBP-α, -β, -γ, -δ, -ε, and -ζ) characterized by a conserved leucine zipper C-terminal domain next to a positively charged DNA-binding domain (34, 35). C/EBP-α specifically induces early myeloid precursors to enter CMP differentiation pathways, whereas C-EBP-ε and Gfi-1 are implicated in the further steps of granulopoiesis, such as in the terminal phase of differentiation (36, 37). In humans, the transit time from HSC to mature neutrophils ranges from 4 to 6 days, after which neutrophils are available for release into the circulation (28, 38).

Once in the circulation, the neutrophil lifespan has been estimated to be less than 24 hours (39). However, in tissues their lifespan could be considerable prolonged, up to 2-4 days (3). In tissues and during an inflammatory process, other cells present in the microenvironment, such as macrophages, can secrete cytokines (e.g., GM-CSF, G-CSF, TNFα) that can prolong the lifespan of neutrophils (40).

The hematopoietic system is capable of rapid adaption to a variety of stimuli, such as stress, bleeding, or infections by increasing the rate of granulopoiesis (41). This program is known as “emergency granulopoiesis” and is characterized by leucocytosis, neutrophilia, and the presence of immature cells in the peripheral blood (42). Different cytokines, including G-CSF, GM-CSF, IL-3 and IL-6 are involved in the activation of emergency granulopoiesis (31, 42).

**Figure 1:**
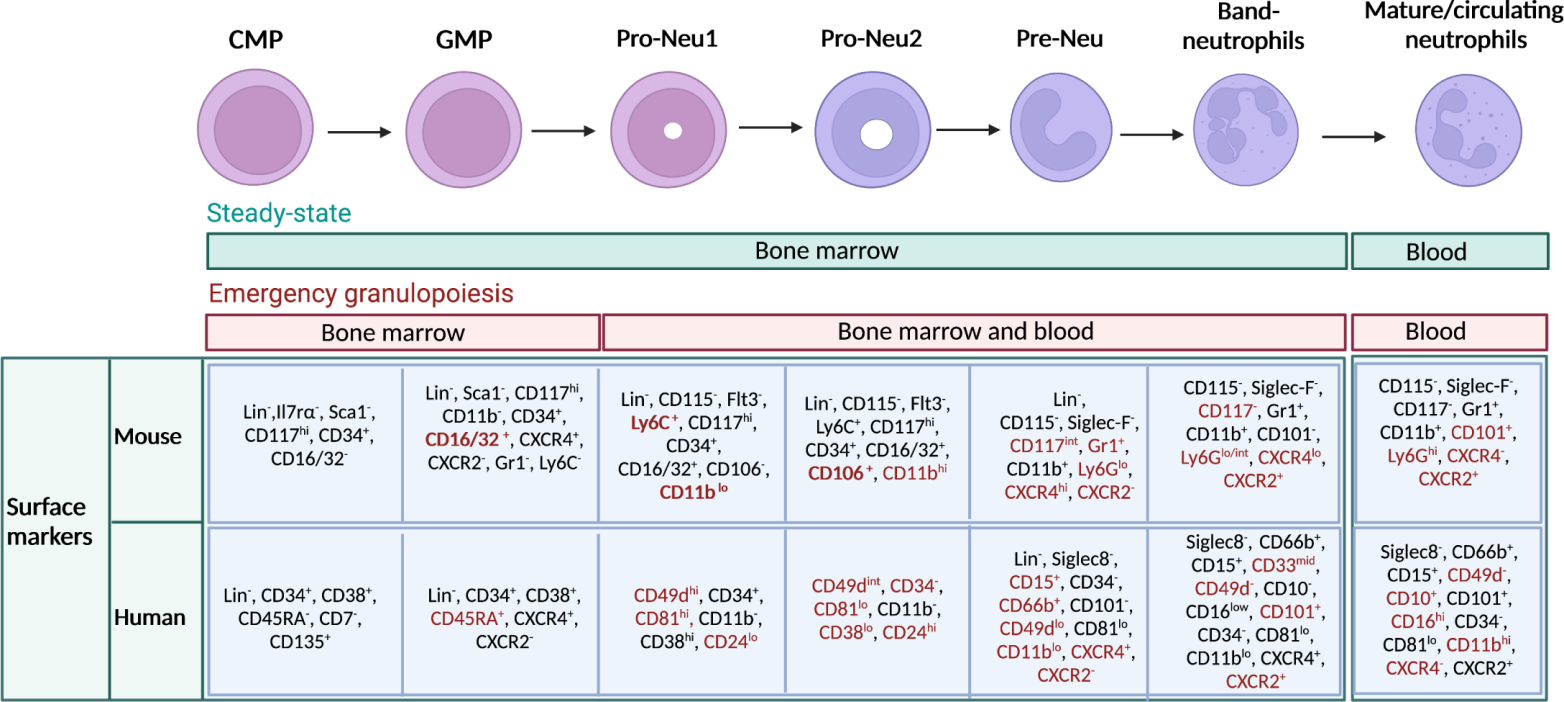
Maturation stages of neutrophils and immunophenotype in mouse and human. Schematic representation of neutrophil developmental stages in the bone marrow (BM), with associated known markers in human and mouse. Neutrophils arise from hematopoietic stem cells (HSCs) in the BM, which give rise to multipotent progenitors, then common myeloid progenitors (CMPs), granulocyte–monocyte progenitors (GMPs), and subsequently to neutrophil-committed precursors. In the BM, GMPs mature into proNeus and then into preNeus, which differentiate into band neutrophils. Finally, non-proliferative mature neutrophils are released in the circulation. Green bar indicates the location of neutrophil progenitors during steady state. Red bar indicates the location where neutrophil progenitors can be found during emergency granulopoiesis. Markers that particularly characterize a stage of differentiation or the transition from one stage to another have been highlighted in red. Gr1 represents a cell marker that includes both Ly6C and Ly6G. However, anti-Gr1 monoclonal antibody reacts strongly with Ly6G. and weakly with Ly6C.

Using cutting-edge technologies such as single-cell RNA sequencing (scRNAseq) and mass-cytometry by time of flight (CyTOF), it has been possible to highlight discrete subsets of neutrophil precursors (4–7). For instance, a population of proliferating neutrophil precursors (defined as preNeu), characterized as Gr1^+^ CD11b^+^ CXCR4^hi^ CD117^int^ CXCR2^-^ cells in mice and with the potential to generate non-proliferating immature which subsequently become mature neutrophils has been recently identified (4). This first observation led to the identification of preNeu progenitors. These progenitors were found both in mice and humans and represent a heterogeneous population of cells described as proNeu1, proNeu2 and preNeu (6). In humans, a recent study identified a population of CD66b^−^CD64^dim^CD115^−^ neutrophil-committed progenitor cells (NCPs) within SSC^lo^CD45^dim^CD34^+^ and CD34^dim/−^ cells found in the BM (43). In **Figure 1** are summarised all neutrophil developmental stages with known associated markers.

### Neutrophil mobilization and recruitment

The regulation of the expression of genes coding for CXCR4 and CXCR2 is essential for the final step of neutrophil maturation and their release in the bloodstream (2, 44). In steady state, stromal cells express CXCL12 in the BM, a ligand for CXCR4, which is expressed on the surface of immature neutrophils and supports their retention in the BM (12, 45). As neutrophils mature, the expression of CXCR4 is downregulated, while the expression of CXCR2 is increased. The abundance of the chemokine CXCL2, which is the ligand for CXCR2, in the circulation triggers the release of neutrophils into peripheral blood (2). A peculiar phenomenon is related to the circadian regulation of CXCR2 and CXCR4 expression. As demonstrated by Adrover José M. et al, the circadian clock transcription factor *Bmal1* drives the expression of CXCL2 to induce CXCR2-dependent diurnal changes in the transcriptional landscape and migration capacity of neutrophils (46). These diurnal alterations are related to neutrophil aging and can be antagonized by CXCR4 signalling (46, 47).

The recruitment of neutrophils in inflammatory sites is initiated by the interactions between circulating leukocytes and activated endothelium. The endothelium can be directly activated by pattern-recognition receptors (PRRs) signalling (48). Neutrophils are primed by exposure to pro-inflammatory cytokines, such as tumour necrosis factor-α (TNF-α), IL-1β and IL-17, chemokines, growth factors, pathogen-associated molecular patterns (PAMPs) and by interacting with the activated endothelium (1, 38, 49). Neutrophils express the chemokine receptors CXCR1 and CXCR2 and the presence of their ligands (CXCL1, CXCL2) provides important chemotactic signalling for neutrophil recruitment into inflamed tissues (50).

Neutrophil recruitment includes the following steps: tethering, rolling, adhesion, crawling, and transmigration (49). Activated endothelial cells upregulate the expression of P-selectin and E-selectin, two molecules that interact with their glycosylated ligands expressed by neutrophils, such as the P-selectin ligand 1 (PSGL-1). The interaction leads to the tethering of neutrophils and their rolling in the direction of the blood flow (20, 51). The activation of neutrophils induces changes in the conformation of cell surface-expressed integrins, which subsequently show higher affinity for their ligands (1). In particular, neutrophils express constitutively high levels of the integrins macrophage-1 antigen (MAC1; CD11b\CD18) and lymphocyte function-associated antigen 1 (LFA1; CD11a\CD18), which undergo conformational changes to bind to the transmembrane glycoproteins endothelial intercellular adhesion molecule 1 (ICAM1) and ICAM2 expressed by endothelial cells (52). Once arrested on the endothelium, neutrophils are ready to transmigrate from the vasculature to the inflammatory site where they can exert different roles, according to tissue context (3, 13). Recent investigations showed that neutrophils can also migrate from inflamed tissues into the peripheral blood through a process known as reverse migration (53). The process of reverse migration has been proposed as a mechanism to prevent excessive inflammation and tissue damage. In the tissue, the elimination of apoptotic or aged neutrophils by macrophages promotes the resolution of the inflammation (see below) (54).

### Neutrophil effector mechanisms

Neutrophils have long been viewed as primary effector cells in the elimination of pathogens and during the acute inflammatory process (55). Thus, their effector mechanisms are classically associated with antimicrobial features. However, their phenotype and function can be different in different inflammatory contexts, such as in fibrosis and cancer.

The antimicrobial potential of neutrophils includes phagocytosis, degranulation, production of reactive oxygen species (ROS) and release of neutrophils extracellular traps (NETs) (1, 56). Additionally, neutrophils secrete a broad spectrum of cytokines and chemokines that can orchestrate the adaptive and innate immune systems (1, 57, 58).

An important mechanism of pathogen elimination consists in the process of phagocytosis, where receptors, such as the FcγR, are used for the engulfment of opsonised or non-opsonised pathogens (59). After the engulfment, a phagosome is formed and its maturation mediates pathogen clearance through the presence of a variety of hydrolytic enzymes and the production of ROS (60). Additionally, neutrophils can eliminate pathogens through the extracellular release of NETs and cytotoxic granules (1). Three types of neutrophil granules have been described: primary (also called azurophilic), secondary (or specific), and tertiary (or gelatinase) granules (61). Primary granules are generally defined by their content of acidic hydrolases, myeloperoxidase (MPO), and microbicidal molecules, such as bactericidal/permeability-increasing protein (BPI) and defensins (28, 61, 62). Specific granules are rich in molecules that participate in neutrophil microbicidal activities, such as lactoferrin, cathelicidin and the long pentraxin 3 (PTX3) (63). Finally, tertiary granules, which are mobilized when neutrophils established primary rolling contact with the activated endothelium, are characterised by matrix-degrading enzymes, such as gelatinase, and membrane receptors including CD11b/CD18, CD67, CD177, fMLF-R, SCAMP, and VAMP2 (64).

Neutrophils can undergo a unique form of cell death, called NETosis, which results in the formation of NETs (59, 65). NETs are large, extracellular, web-like structures composed of cytosolic and granule proteins that are assembled on a scaffold of decondensed chromatin (65, 66). These structures are extruded by activated neutrophils and pathogens are immobilized and killed through the exposure to high concentrations of effector proteins, including enzymes and antimicrobial molecules (e.g., defensins and cathelicidins) (63, 65).

Neutrophils are also able to synthesize different mediators that can orchestrate both innate and adaptive immunity (67). For instance, neutrophils produce pro- and anti-inflammatory cytokines (e.g., IL-6, TNF-α, IL-1β, IL-1RA, TGFβ) and chemokines (e.g., CXCL8, CXCL10) (60, 67–69).

## Neutrophil functional heterogeneity

The classic view of neutrophils as poorly adaptive cells has been challenged by recent findings. Indeed, a growing number of studies have demonstrated the heterogeneity and plasticity of neutrophils. It has been reported that circulating and tissue neutrophils displayed substantial heterogeneity, in terms of phenotype, maturation\activation states and effector functions, due to circadian oscillations, aging, and the presence of microenvironment cues (3, 8, 70). Accordingly, freshly released and aged neutrophils showed considerable diversity in their repertoire of chemokines, PRRs, adhesion molecules, granule proteins, and in their ability to release NETs (46, 71–73). These results support the hypothesis that neutrophil-dependent immune response is not stable during the day and may be dependent on environmental cues (74).

Neutrophils are recruited in healthy tissues and can contribute to their physiological functions (3, 47, 75–78). Indeed, a recent report showed that neutrophils can acquire tissue-specific transcriptional programs that support tissue homeostasis in different organs. In the lung, this process is driven by the CXCL12/CXCR4 axis (3). Accordingly, the lack of CXCR4 in neutrophils impaired their acquisition of the lung-specific signature (3). Neutrophils are present in other tissues, such as in the intestine, white adipose tissue, skin, and skeletal muscle but their role and functions need to be fully elucidated.

Neutrophils with specialized functions were reported in the BM. Indeed, BM-resident neutrophils are important to sustain the regenerative capacity of medullary sinusoid via the production of TNFα (79). In addition, mature BM neutrophils expressing the histidine-decarboxylase were reported to support the maintenance of the quiescence state of HSCs, through the release of histamine and consequent activation of the H_2_ receptor on HSC (80). Finally, BM neutrophils can secrete prostaglandin E2 (PGE2), which stimulates the production of osteopontin by preosteoblasts and the retention of HSCs (81).

Specialized neutrophils were also found in the marginal zone of the spleen, where they can promote the immunoglobulin class switching and production of antibodies by activated B cells through the production of B-cell activating factor (BAFF), a proliferation-inducing ligand (APRIL) and IL-21 (78). In lymph nodes, MHCII^+^ neutrophils were found in the proximity of T and NK cells and are thought to promote T cell activation (76).

## Neutrophils in systemic inflammation

**Figure 2:**
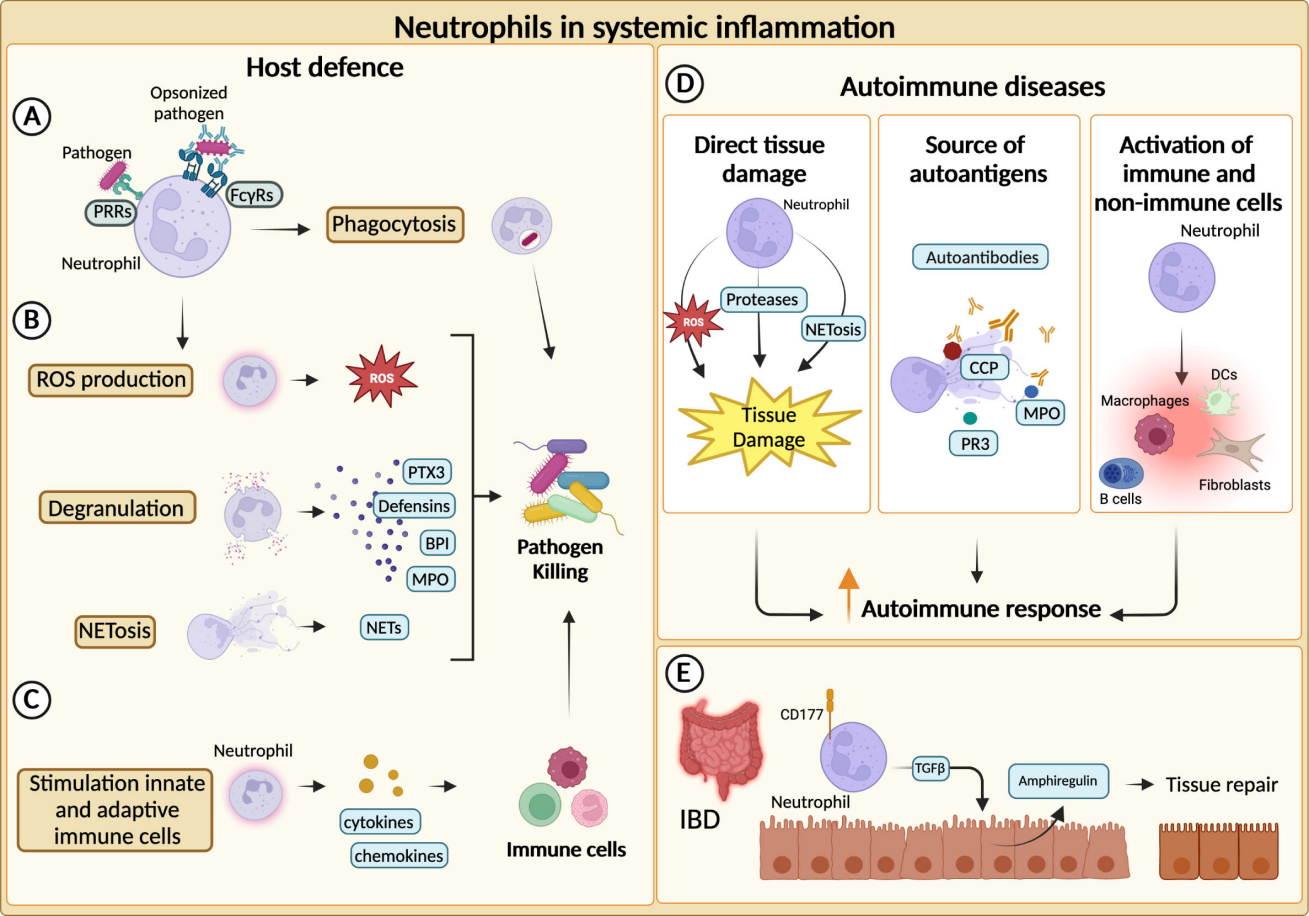
Neutrophils in systemic inflammation. **(A)** Neutrophils phagocytize and kill pathogens by using Fcγ receptors (FcγRs) and pathogen recognition receptors (PRRs) for the engulfment of opsonized or non-opsonized pathogens. **(B)** Neutrophils can eliminate pathogens through degranulation, production of reactive oxygen species (ROS) and release of neutrophil extracellular traps (NETs). **(C)** Neutrophils actively recruit and activate other innate and adaptive immune cells ensuring a complete clearance of the pathogens. **(D)** Neutrophils can fuel the autoimmune response and exacerbate tissue-damaging inflammation through various mechanisms. First, they can directly cause tissue damage. NETs-associated molecules, such as cyclic citrullinated peptides (CCP), myeloperoxidase (MPO) and proteinase 3 (PR3), can represent an important source of autoantibody targets. Neutrophils promote autoimmune response by recruiting and activating adaptive and innate immune cells as well as non-immune cells. **(E)** In inflammatory bowel diseases (IBD), CD177^+^ neutrophils producing TGFβ stimulate the production of the tissue repair-promoting factor amphiregulin (AREG) by intestinal epithelial cells and promote tissue repair.

Neutrophils represent the first line of defence against invading pathogens and are recruited to the site of infection and inflammation by chemokines secreted by host cells and pathogen-derived products (12). Thereafter, they can efficiently kill invading pathogens and actively recruit other immune cells to complete the clearance of foreign microorganisms (1). Neutrophils are equipped with a broad range of antimicrobial weapons to clear pathogens through the combination of different effector mechanisms including, phagocytosis, production of ROS, degranulation, and release of NETs (59) In addition, neutrophils express phagocytic receptors and a set of soluble PRRs with an opsonic activity which are considered as functional ancestors of antibodies (82) (**Figures 2A–C**).

Given their extraordinarily potent effector functions, excessive or uncontrolled neutrophil activation could have deleterious consequences for the host. An increasing body of evidence suggests that an abnormal neutrophil activation might play a prominent role in the pathophysiology of severe inflammation-related disorders, such as Coronavirus disease 2019 (COVID-19) (83, 84). Indeed, severe COVID-19 has been associated with increased neutrophil-to-lymphocyte ratio (85) and concentration of neutrophil-related molecules in the serum (e.g., calprotectin, NETs-associated molecules) (86–88). Therefore, neutrophilia is associated with bad prognosis in COVID-19 patients (84).

A deleterious role for neutrophils has been observed also in the context of autoimmune diseases and chronic inflammation. Diseases such as systemic lupus erythematosus (SLE), rheumatoid arthritis (RA), vasculitis, type I diabetes (T1D), multiple sclerosis (MS), idiopathic inflammatory myopathies (IIM) are characterized by the loss of immune tolerance to self-antigens. Although autoimmune diseases have been mainly ascribed to dysfunctions in adaptive immune response, an increasing number of evidence points to a possible role for innate immunity and in particular neutrophils (17, 89–91).

Increased neutrophil infiltration or augmented levels of neutrophil-specific molecules are often found within inflamed tissues of both SLE and IIM patients (92, 93). Moreover, neutrophil-related biomarkers (e.g., neutrophil serine proteinases, calprotectin, NETs) correlate with clinical parameters and response to therapy in various disease settings, further corroborating the role of neutrophils in autoimmunity (94–98). In autoimmune diseases, neutrophils can exacerbate tissue-damaging inflammation through a variety of mechanisms, including the production of ROS, proteases and the activation of immune and not immune cells (17, 99) (**Figure 2D**). Neutrophils from SLE patients showed cytotoxic activity towards endothelial cells *in vitro*. Consistently, SLE patients present an augmented risk of endothelial dysfunction (92, 100). Similarly, NETs have been found to affect the function of myoblast in IIM patients and can contribute to the cleavage of articular cartilage in RA (100). Interestingly, different components of NETs (e.g., double-stranded DNA, histones, citrullinated peptides (CCP), MPO and proteinase 3 (PR3)) are well-described targets of autoantibodies in autoimmune diseases (96, 97, 101, 102) (**Figure 2D**).

Neutrophils are endowed with the ability to recruit and activate a plethora of cells including adaptive and innate immune cells as well as non-immune cells. Thus, they can contribute to nourishing autoimmune responses and worsening tissue damage. In SLE, it has been described that NET-LL37-DNA complexes activated autoreactive B cells via TLR9, thereby stimulating autoantibody production (103).

Altogether, these mechanisms could explain how neutrophils can fuel autoimmune responses. However, neutrophils have also shown healing potential in some inflammatory diseases (104, 105). Indeed, the role of neutrophils in inflammatory bowel disease (IBD) appears to be paradoxical, as both beneficial and detrimental roles have been reported (23). An elevated number of circulating neutrophils has been reported in patients with IBD and can be associated with the severity of the disease (106). Similar observations were made for neutrophil-dependent mediators (107–109). Although unrestricted activation of neutrophils could trigger tissue damage and inflammation, neutrophils have been reported to be essential to maintain gut homeostasis (23). For instance, in a mouse model of colitis, neutrophils can contribute to the resolution of pathology through the production of TGFβ, which increases the levels of amphiregulin in intestinal epithelial cells (110). Moreover, neutrophils have been proposed to be a producer of IL-22, which is known to be protective in IBD (26). Consistently, a population of CD177^+^ neutrophils producing IL-22 and TGFβ negatively regulates IBD (25) (**Figure 2E**).

### Neutrophil heterogeneity in systemic inflammation

Higher demand for neutrophils during systemic inflammation activates a process known as emergency granulopoiesis (see also above) (42). This phenomenon leads to the release in the circulation of immature neutrophils along with mature neutrophils, generating a mingling of populations with different features, including immunosuppressive properties (111). A part of this heterogeneous population has been empirically called low-density neutrophils (LDNs), based on sedimentation properties, to distinguish them from normal-density neutrophils (NDNs). Indeed, after density gradient sedimentation, LDNs are found in the peripheral mononuclear cell (PMBC) layer while NDNs are found on the top of the erythrocytes layer (111).

LDNs have been found in the peripheral blood of patients with a broad variety of acute and chronic inflammatory diseases, including cancer, HIV-1 infection, SARS-CoV-2 infections, sepsis, graft-versus-host disease (GvHD), trauma, or in healthy donors who received G-CSF treatment for stem cell mobilization, or hematopoietic stem cell transplantation (HSC-T) (112–114).

The presence and phenotype of the LDN population in these different contexts have been exhaustively reviewed (111, 112), but there are still open questions. Firstly, there are no specific markers for LDNs and in humans these cells are typically described as CD66b^+^ CD15^+^ CD14^-/dim^ CD33^dim^ HLA-DR^-^, a phenotypic profile identical to the one of NDNs (112). The lack of specific markers to distinguish LDNs from NDNs led to confusion and contrasting reports in the past decade (115). Recently, CD98 has been proposed as a marker of LDNs found in patients treated with G-CSF and can identify a subset of LDNs in SLE patients (116). CD98^+^ LDNs of SLE patients produced more proinflammatory cytokines and chemokines than NDNs and were resistant to apoptosis (116). Accordingly, the presence of CD98^+^ LDNs correlated with disease activity in SLE patients. Interestingly, the inhibition of CD98 reduced the metabolic flexibility of this population limiting their pathogenic capacity, thus identifying CD98 as a potential therapeutic target (116).

LDNs are generally endowed with immunosuppressive properties. Accordingly, these cells express Arginase 1 (Arg1), ROS or programmed cell death ligand 1 (PDL-1), all molecules involved in T cell suppression (115). However, neutrophils with proinflammatory properties in the LDN fraction have been reported in patients with SLE or RA (92, 100). Conversely, neutrophils with immunosuppressive features have been observed within NDNs (111, 117). Therefore, the functional properties of total LDN and NDN fractions remain redundant, and the contradictory reports could be the result of neutrophil heterogeneity.

LDNs represent a heterogeneous population of cells which can include the entire spectrum of neutrophil differentiation stages (11). Accordingly, LDNs can have different maturation stages and different functional properties. CD10 has been proposed as a marker to discriminate mature from immature neutrophils in inflammatory conditions. In G-CSF treated donors, CD10 identified two populations of LDNs with different immunological features (118). Accordingly, CD66b^+^ CD10^-^ neutrophils were able to promote T cell survival, proliferation and IFNγ production, whilst CD66b^+^ CD10^+^ cells were shown to suppress T cell proliferation and IFNγ production in a CD18-mediated contact-dependent Arg1 release (118).

The extraordinary heterogeneity of LDNs could also be related to the fact that these cells are involved in different pathological contexts, spanning from infection to inflammatory disorders and HSC-T. Thus, it is conceivable that neutrophils are shaped by the specific inflammatory context giving rise to a spectrum of activation states.

A step toward a better understanding of the mechanisms allowing neutrophil diversity in humans has been recently achieved (11). The authors uncovered the diversity of human neutrophils in different settings, including G-CSF treatment, HSC-T, pancreatic ductal adenocarcinoma (PDAC) and SARS-CoV-2 syndrome. By combining multiparametric immunophenotyping, quantification of plasma cytokines, transcriptomic and computational analysis, they showed that stress-elicited neutrophils underwent profound changes in the expression dynamics of developmental genes, and they concomitantly acquired stimulus-specific gene signatures (11). Interestingly, the extent and the type of transcriptional response were dependent on neutrophil maturation stage, with the strongest dynamics observed in differentiated neutrophils. In the blood of patients receiving HSC-T, an acute IFN response has been identified. Interestingly, this response is translated into a significant upregulation of IFN-stimulated genes in neutrophils (11). These findings underlined the transcriptional plasticity of neutrophils in response to environmental cues and open to the possibility to use neutrophil transcriptome features as a biomarker.

## Neutrophils in the resolution of inflammation and tissue repair

Tissue repair represents the physiological reaction that restores tissue homeostasis upon injury. The elimination of apoptotic neutrophils by macrophages contributes to the resolution of inflammation (119, 120). Efferocytosis not only eliminates dying neutrophils avoiding pro-inflammatory secondary necrosis but also actively induces a pro-resolving phenotype in macrophages. Macrophages after efferocytosis show increased production of anti-inflammatory cytokines (e.g., TGF-β and IL-10), decreased expression of pro-inflammatory mediators (e.g., TNFα, IL-12, IL-1β and IL-6) and increased biosynthesis of pro-resolving lipid mediators, including resolvin D1 (RvD1), RvD2, and RvE2 (22, 121). Beyond efferocytosis, additional pro-resolving neutrophil-mediated mechanisms have emerged. Apoptotic neutrophils express high levels of CCR5 that scavenges chemokines preventing further recruitment of inflammatory cells (122). Moreover, during the initial phases of inflammation, neutrophils can undergo a lipid mediator class switch upon PGE2 stimulation. The production of leukotrienes, which support inflammation, is shifted towards the synthesis of lipoxins (LX), which favor resolution by dampening the recruitment of neutrophils to the site of inflammation (123). Furthermore, extracellular vesicles released either by living or apoptotic neutrophils induce the production of pro-resolving lipid mediators and anti-inflammatory cytokines by macrophages, increasing their efferocytosis capacity (121). The protein annexin 1 (AnxA1) found expressed on neutrophil-derived microparticles has been implicated in this anti-inflammatory effect (124).

## Neutrophils in cancer

Neutrophils have been found to infiltrate solid tumours, including non-small cell lung cancer (NSCLC), colorectal cancer (CRC), gastric cancer, hepatocellular carcinoma, melanoma, breast cancer, renal carcinoma, and sarcomas (9, 13, 21, 125–129). Although tumour infiltrating neutrophils (TANs) have been commonly associated with a poor prognosis, with few exceptions, the overall contribution of neutrophils in cancer is still unclear (9, 13).

### Neutrophils in tumour promotion

Neutrophils can favour tumour development through direct mechanisms, by promoting genetic instability and cell proliferation, or by indirect mechanisms such as through the promotion of the metastatic spread or the inhibition of the anti-tumour immune response (130).

**Figure 3:**
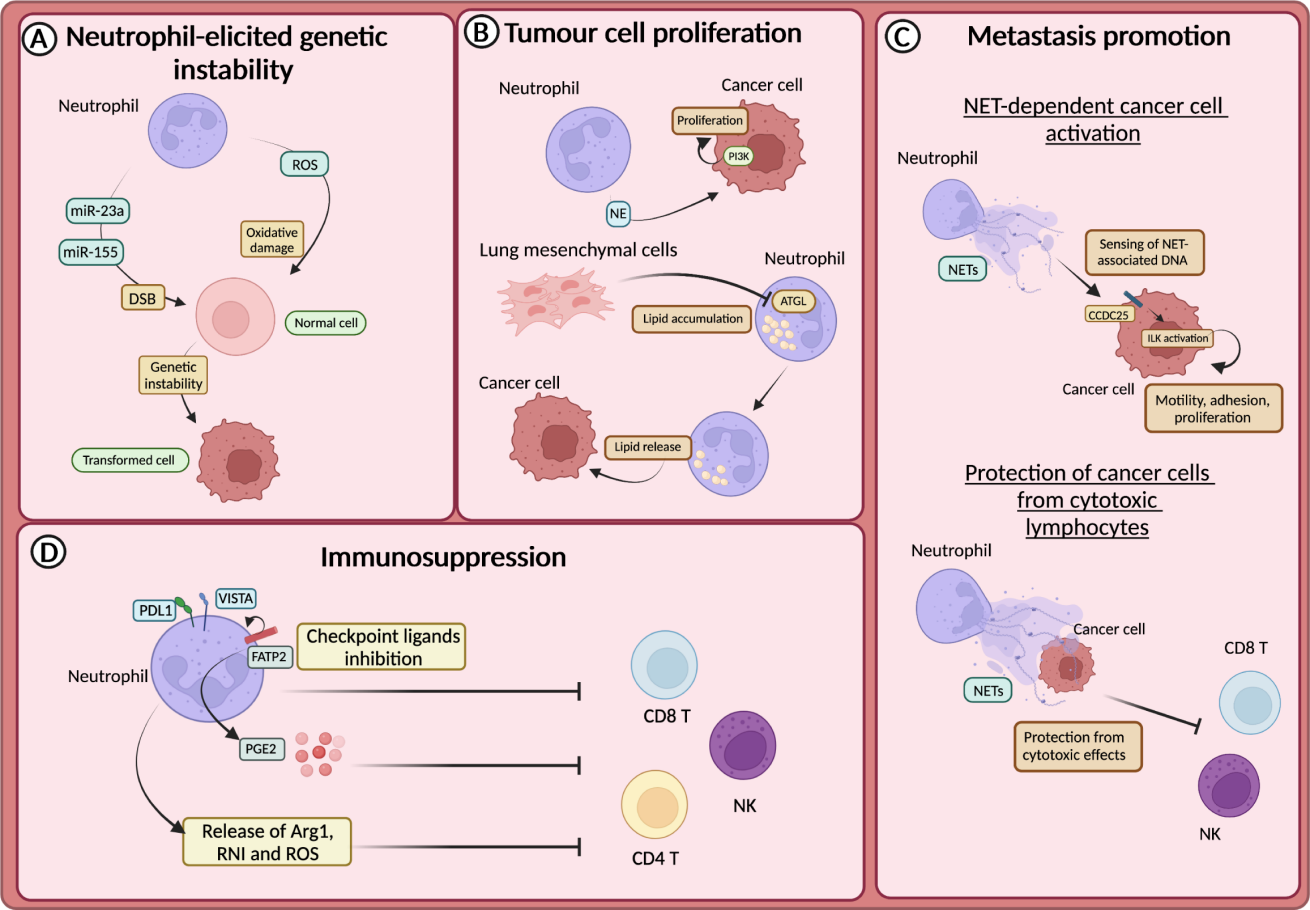
Neutrophils in tumour promotion **(A)** Neutrophil-derived reactive oxygen species (ROS) or miR23a and miR-155, by promoting the accumulation of double strand breaks (DSB), sustain genetic instability of cells. **(B)** Neutrophils sustain the proliferation of tumour cells through the release of neutrophils elastase (NE), and subsequent activation of PI3K. In the premetastatic niche, lung-resident mesenchymal cells forced neutrophils to accumulate lipids through the inhibition of the adipose triglyceride lipase (ATGL). Lipids are then released to feed tumour cells. **(C)** CCDC25 on tumour cells acts as a sensor for neutrophil extracellular trap (NET)-associated DNA and activates the integrin-linked kinase (ILK) signalling, promoting liver metastasis formation. NETs protect cancer cells from the cytotoxic activity of NK and CD8^+^ T cells. **(D)** Neutrophils drive immunosuppression and the dysfunction of T cells in various ways, including the expression of lymphocytes checkpoint (e.g., PD-L1 and VISTA), the production of ROS, arginase 1 (Arg1) or reactive nitrogen intermediate (RNI) and through fatty acid transporter protein 2 (FATP2)-dependent production of prostaglandin E2 (PGE2).

The promotion of genetic instability by neutrophils was firstly demonstrated in lung cells and linked to the production of ROS (131). This first observation has been confirmed by other studies, including in intestinal and lung cancer models, in which neutrophils have been suggested to induce tissue damage and genetic instability in a ROS-dependent manner (132, 133) (**Figure 3A**). In addition to ROS, recent findings showed that neutrophils promoted the accumulation of double-strand breaks (DSB) in the injured epithelium through the release of microRNAs (miR-23a and miR-155) (134) (**Figure 3A**).

Neutrophils produce several mediators, including cytokines and growth factors that can fuel tumour growth (21, 135). It has been reported that mediators derived from neutrophil granules were involved in tumour promotion (136–138). For instance, NE can activate the proliferation of different cancer cells, including human oesophageal cell lines, mammary epithelial cells, human prostate cancer cells and human and mouse lung cancer cells (136–138). In this regard, it has been demonstrated that NE induced the degradation of the insulin receptor substrate 1 (IRS-1), which inhibits the phosphoinositide 3-kinase (PI3K) (**Figure 3B**). Thus, PI3K can interact with the PDGF receptor (PDGFR) and induce the proliferation of human and mouse lung cancer cells (136). Accordingly, in a model of lung adenocarcinoma driven by *kras* mutation, NE-deficient mice showed reduced tumour development (136).

Neutrophil metabolic reprogramming is another fascinating mechanism through which neutrophils feed tumour cells and promote metastasis (139). In the pre-metastatic niche, lung-resident mesenchymal cells co-opted neutrophils to accumulate neutral lipids via the repression of the adipose triglyceride lipase (ATGL). In turn, the transfer of lipids from neutrophils to tumour cells via a micropinocytosis-lysosome pathway dramatically potentiates their proliferative and survival capacity and metastatic potential (139) (**Figure 3B**).

The pro-metastatic activity of neutrophils is also achieved through the promotion of angiogenesis, the protection of circulating tumour cells (CTCs), the activation of dormant cancer cells and/or the recruitment of tumour cells in the pre-metastatic niche (140–142). For instance, neutrophil-derived mediators, such as Bv8 and MMP-9 are known for their pro-angiogenetic properties (141).

Clusters of neutrophils and CTCs were observed in the circulation of patients with breast cancer and mouse cancer models (143). Tumour cells within the cluster of neutrophil-CTCs displayed increased proliferative capacity and were more efficient in metastasis formation (143).

The release of NETs has long been associated with metastasis promotion. NETs have been observed in different types of tumours and participate in tumour-promoting inflammation, angiogenesis, extracellular matrix remodelling and proliferation of tumour cells (144–151). Several studies showed that NETs could support the formation of metastasis by entrapping CTCs and facilitating their seeding and proliferation in a distant anatomical site (144, 145). NETs can also act as a recruiting signal for CTCs expressing the coiled-coil domain containing protein-25 (CCDC25), which acts as an extracellular DNA sensor and can activate cell motility (151). Indeed, the engagement of CCDC25 by NET-associated DNA fostered the integrin-linked kinase (ILK) pathway leading to increased adhesion, motility, and growth of metastatic cancer cells in the liver (**Figure 3C**). Accordingly, the formation of metastasis in the liver and in the lung through this mechanism is reduced in CCDC25-deficient mice (151). NETs can also serve as a shield for CTCs and protect them from the cytotoxic activity of CD8^+^ T cells and natural killer (NK) cells (149). Notably, pharmacological inhibition of NETs formation synergized with anti-PD1 plus anti-CTLA4 treatment (149) (**Figure 3C**).

Neutrophils have long been associated with the suppression of the immune response, which can occur in several ways (152). For instance, neutrophils produce mediators that suppress the activity of T cells, such as Arg1, ROS, reactive nitrogen intermediate (RNI), and PGE2 (130) (**Figure 3D**). Endoplasmic reticulum (ER) stress associated with lipid metabolism alteration characterized neutrophils with immunosuppressive features (153). The expression of proteins involved in lipid trafficking and metabolism such as LOX-1, CD36 and fatty acid transport protein 2 (FATP2) were expressed by immunosuppressive neutrophils (154). In non-small cell lung cancer (NSCL) patients, neutrophils with ER stress response were observed (155). The activation of ER response induced the expression of LOX-1 on neutrophils, which are also characterized by high production of ROS and Arg1 production (155). In addition, FATP2 expression on neutrophils induced the biosynthesis of PGE2 and consequently an immunosuppressive activity (155). Indeed, PGE2 has been shown to impair cytotoxic activity and survival of NK cells and CD8^+^ T cells (156, 157) (**Figure 3D**).

The immunosuppressive activity of neutrophils can also be achieved by direct inhibition of T cells through the engagement of checkpoint molecules. Neutrophils expressing the programmed cell death 1 ligand 1 (PD-L1) or the V-domain immunoglobulin suppressor of T-cell activation (VISTA) were reported in human and mouse models of hepatocellular carcinoma, melanoma, and gastric cancer (158–162). In a murine model of melanoma, the blockade of VISTA induced a potent pro-inflammatory response in myeloid cells and reduced their immunosuppressive potential (162) (**Figure 3D**). However, the treatment has no effect on neutrophil-dependent immunosuppression, suggesting the role of VISTA on neutrophils could be different from other myeloid cells and requires further investigation.

### Neutrophils in the anti-tumour response

**Figure 4:**
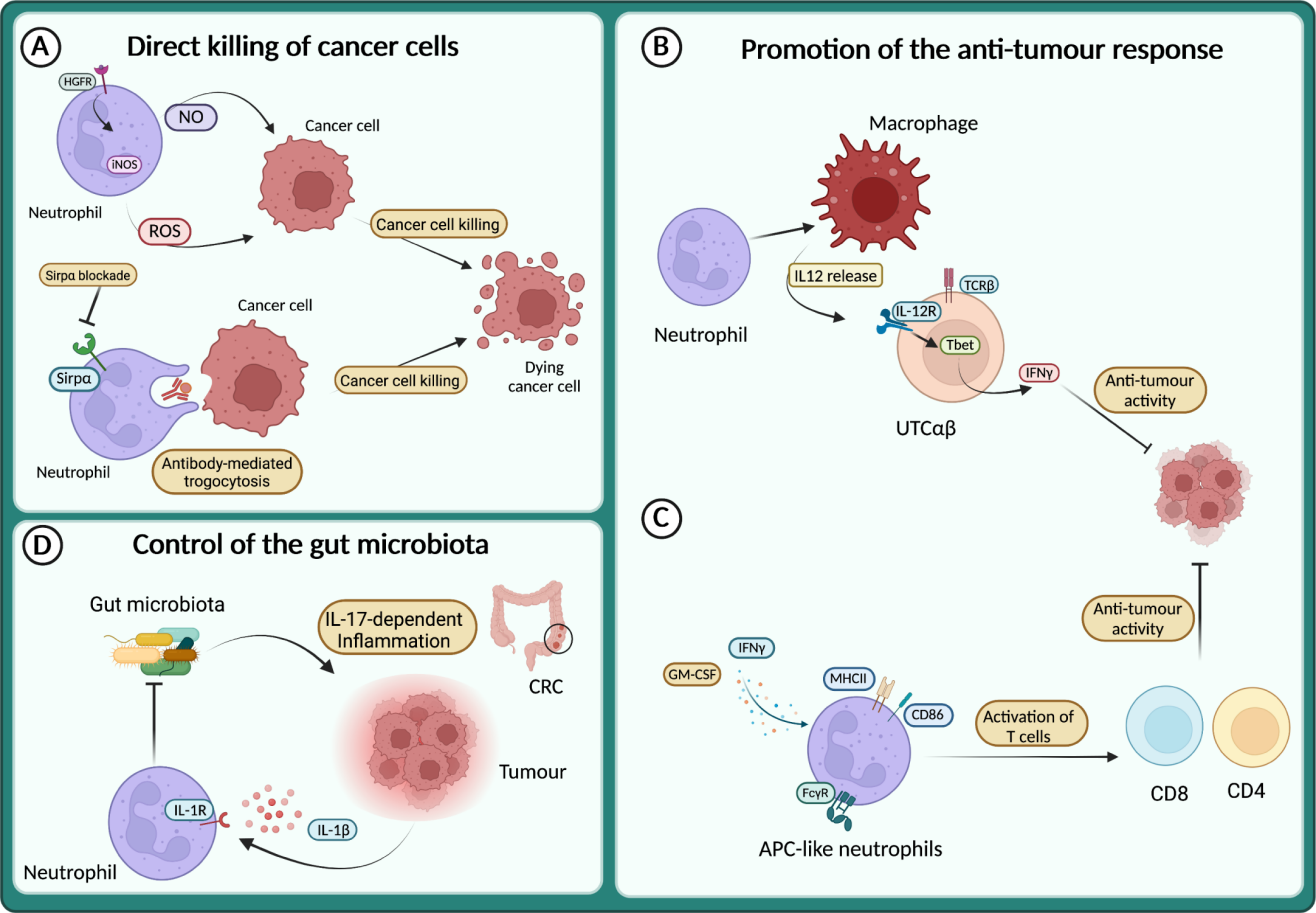
Neutrophils in the anti-tumour response **(A)** Neutrophils directly kill cancer cells through the release of reactive oxygen species (ROS) and NO or via antibody-mediated trogocytosis of antibody-opsonized cancer cells. **(B)** Neutrophils sustain IL-12 production by macrophages, which in turn activates a subset of unconventional αβ T cells (UTCαβ) to produce IFNγ and suppress tumour growth. **(C)** APC-like neutrophils can be induced in response to GM-CSF and IFNγ, or by FcγR-mediated endocytosis of antibody-antigen and can activate T cell anti-tumour response. **(D)** In response to IL-1β neutrophils restrict the invasion of gut microbiota and thus dampen microbiota-dependent inflammation in the context of colorectal cancer (CRC).

Although most of the data from patients and mouse models support the pro-tumour role of neutrophils, a growing number of studies describe their anti-tumour activity (127–129, 163–165). It has long been known that neutrophils can directly kill tumour cells through the release of ROS or NO (135, 166–168) (**Figure 4A**). Neutrophils expressing TNF-related apoptosis-inducing ligand (TRAIL) displayed enhanced tumour cell killing *in vitro* in an IFNγ-dependent manner (169). Interestingly, tumour cell derived TNFα induced the expression of the hepatocyte growth factor receptor (HGFR or Met) on neutrophils, which in response to HGF expressed the inducible nitric oxide synthase (iNOS) and NO to kill cancer cells (170) (**Figure 4A**). Conversely, others have reported that HGF-Met signalling in neutrophils induced an immunosuppressive phenotype associated with the inhibition of T cells and reduced response to adoptive T cell transfer and checkpoint blockade therapies (171). Therefore, the role of Met and HGF on neutrophils remains to be fully elucidated.

The direct killing of cancer cells by neutrophils can also be achieved through antibody-dependent cellular cytotoxicity (ADCC). Indeed, neutrophils can induce cell lysis via antibody-mediated trogocytosis of antibody-opsonized cancer cells (**Figure 4A**). Interestingly, blocking the CD47-Sirpα interaction enhanced cancer cell killing, suggesting that this pathway can be targeted to potentiate neutrophil cytotoxic activity (172). Recently, a cocktail consisting of TNFα, CD40 agonist and a tumour-specific antibody was shown to increase the ability of neutrophils to kill human tumour cells *in vitro* (173). In mouse models, the same combination stimulated the anti-tumour activity of neutrophils, leading to the eradication of established tumour and reduction of metastasis (173).

The neutrophil killing capacity can be potentiated also by the deletion of the atypical chemokine receptor 2 (ACKR2). Indeed, in models of breast cancer metastasis, genetic ablation of Ackr2 resulted in increased mobilization of myeloid cells, including neutrophils endowed with increased ROS-mediated cytotoxic activity (174).

Neutrophils can also act as triggers of the immune response against tumours, by recruiting and fostering the activity of other immune cells. For instance, neutrophils produce chemokines that recruit T cells or other leukocytes such as CXCL1, CXCL2, CXCL10, CCL2 and CCL3 (1). Moreover, neutrophils can engage crosstalk to activate T cells, as reported in the model of 3-methilcolantrene induced carcinogenesis (129). Here, neutrophils were essential to promote the production of IL-12 by macrophages, which in turn activated the anti-tumour activity of a subset of unconventional αβ T cells (UTCαβ) (**Figure 4B**).

Interestingly, a mechanism by which neutrophils can counteract carcinogenesis is represented by the acquisition of antigen-presenting cell (APC)-like phenotype. This APC-like behaviour of neutrophils has been observed in NSCL patients, where a subset of immature neutrophils (CD11b^+^ CD15^hi^ CD10^–^ CD16^int/low^) acquired the expression of MHCII and CD86 in response to GM-CSF and IFNγ and triggered a T cell-mediated anti-tumour response (175) (**Figure 4C**). Notably, FcγR-mediated endocytosis of antibody-antigen complexes converts neutrophils into a more potent APC-like cells (176) (**Figure 4C**). A more recent report showed the dual role of neutrophils in tumour-draining lymph nodes (LN) during head and neck cancer (HNC) progression (177). In these settings, neutrophils transmigrate and shape T cells activation in a stage-dependent manner, with neutrophils acquiring APC-like features and promoting T cell anti-tumour activity in metastasis-free patients, while at a later stage, neutrophils acquire PD-L1 expression and suppress T cell activation. Interestingly, the presence of neutrophils in LN can be used as a prognostic marker depending on the stage of the patients. In transplantable mouse models of cancer (i.e., lung adenocarcinoma and colorectal cancer), a recent study showed that neutrophils with anti-tumour properties were accumulated in tumours during successful immunotherapy with anti-PD-1 and anti-CD40 (178). In treated mice, the expanded clusters of neutrophils were characterized by a *Sell^hi^* phenotype and an interferon-stimulated gene signature. Of note, the neutrophil response requires the transcription factor IRF1 and a loss of its expression led to the failure of the treatment (178). In addition, another study showed that following melanoma-specific CD4^+^ T cell therapy combined with OX40 co-stimulation or anti-CTLA-4, complete tumour eradication was dependent on neutrophils killing of antigen-negative tumour cells. Interestingly, massive neutrophil activation was observed in mouse tumours and in biopsies from melanoma patients treated with immune checkpoint blockade (179).

The role of microbiota in cancer development is emerging and neutrophils have been involved in the control of microbiota in tumour context, including metastasis formation and response to therapy (180–184). For example, in a genetic mouse model of neutrophil deficiency (e.g., *LysM^Cre^;Mcl1^fl/fl^*), enhanced intestinal tumour formation and IL-17-dependent inflammation due to increased intra-tumour bacteria have been reported (164) (**Figure 4D**). On the same line, blocking *Il1r1* in neutrophils resulted in limiting their antibacterial potential and led to increased bacterial invasion of tumours and increased inflammation and cancer progression (165) (**Figure 4D**). In apparent contrast, in a lung cancer model, airway microbiota activated neutrophil release of cytokines. In turn, neutrophil-released cytokines induced the activation and proliferation of lung-resident γδ T cells that promoted inflammation and tumour growth (185).

### Cancer-dependent neutrophil plasticity and heterogeneity

The results discussed above underlined the dual role of neutrophils in cancer. These controversial findings may be ascribed to the plasticity and the heterogeneity of neutrophils in the tumour context (9, 70). Indeed, a growing body of evidence challenged the view of neutrophils as short-lived effectors and poorly adaptive cells.

Cancer-dependent neutrophil perturbations, such as tumour-induced emergency granulopoiesis, are often observed during tumour progression (186–188). Indeed, neutrophil differentiation and maturation trajectories are dramatically altered in tumour-bearing mice and cancer patients, and multiple studies have reported the premature release of early neutrophil precursors and their progenitors into the peripheral blood (4, 6, 189). Neutrophilia can occur through the activation of the IL-17/G-CSF pathway. In a mouse model of breast cancer in KEP mice (i.e., *K14^cre^; Cdh1^fl/fl^; Trp53fl/fl*) secretion of WNT ligands induced IL-1β release from macrophages and induced the production of IL-17 by γδT cells (186, 188). IL-17 enhanced the production of G-CSF and the accumulation of neutrophils in the peripheral blood and in the lung.

Tumour-associated alterations of neutrophil metabolism, which are frequently associated with an immunosuppressive capacity, have been recently reported (see also above). The metabolic activity of neutrophils is generally based on glycolysis for their survival and function and a role for metabolic alterations in neutrophils is emerging in cancer (190). In a model of breast cancer, tumour-elicited neutrophils engage oxidative mitochondrial metabolism to maintain ROS production and suppress T cells (187). On the same line, a subset of LDNs found in the circulation of cancer patients was shown to promote liver metastasis by undergoing a metabolic shift (191). As discussed above, neutrophils in the pre-metastatic lung accumulate neutral lipids upon interaction with resident mesenchymal cells and consequently feed cancer cells by releasing the accumulated lipids (139).

The plasticity of neutrophils in cancer has been demonstrated in response to tumour derived TGFβ, which drives the polarization of neutrophils towards an immunosuppressive phenotype. In this activation state, called N2 to mirror the M1/M2 paradigm of macrophages, neutrophils produced proangiogenic factors (e.g., MMP-9) and inhibited CD8^+^ T cells activation through secretion of Arg1 (192). Conversely, N1 neutrophils were endowed with anti-tumour properties, including a cytotoxic activity towards tumour cells and the promotion of T cell recruitment and activation.

Another level of complexity of neutrophils heterogeneity in cancer is represented by myeloid-derived suppressor cells (MDSCs), which can be immature or mature myeloid cells (e.g., neutrophils and monocytes) endowed with immunosuppressive properties and are found in the circulation, the primary tumour, and metastases (193). MDSCs are functionally characterized by the ability to suppress T cell proliferation and activation *in vitro* (155). However, the phenotypic characterization of these cells is still controversial (194). Indeed, granulocyte MDSCs (G-MDSCs or PMN-MDSCs) are described as CD15^+^ CD66b^+^ CD33^dim^ HLA-DR^-^ in humans and as CD11b^+^ Ly6G^+^ CD84^+^ in mice, making them indistinguishable from other neutrophil subsets (193). Recently, a scRNAseq analysis performed on neutrophil subsets from tumour-bearing mice identified three populations of neutrophils: classic neutrophils, PMN-MDSCs and activated PMN-MDSCs, which showed potent immunosuppressive activity (195). PMN-MDSCs and activated PMN-MDSCs are found in tumours at early stages of tumorigenesis and acquired the expression of CD14, suggesting the use of this marker to distinguish classic neutrophils from PMN-MDSCs in mice.

PMN-MDSCs and N2 neutrophils share several characteristics, including phenotype and morphology, suppression of T cell activation and have been found in conditions of chronic inflammation, including cancer (161, 196–199). Therefore, the description of the different features of these two populations has become a new challenge in the study of neutrophil biology within tumour. Given their strict definition as immunosuppressive cells, we recommend the designation of MDSCs for populations with proven immunosuppressive activity.

## Conclusions and perspectives

Neutrophils are central mediators of the innate immune response. Previously considered to be only a primary defence against invading pathogens, neutrophils have emerged as key players in many inflammatory conditions and immune-mediated diseases, including in cancer.

The role of neutrophils in these pathological contexts is often controversial, as they can exert both beneficial and detrimental functions. This dichotomous behaviour emerged clearly in the context of inflammatory diseases and cancer.

The implementation of high-throughput technologies, such as high-dimensional transcriptomic and epigenomic, and multi-dimensional cytometry, allowed the study of neutrophils at single-cell resolution. These gave rise to numerous studies revealing the heterogeneity and plasticity of neutrophils from their developmental to their effector functions. Thanks to these findings, we are now appreciating how neutrophils can rapidly respond to changes in the environment and adapt their behaviour.

Targeted therapies and immunotherapies are now the frontlines of modern medicine. In this context, it would be beneficial to develop new approaches to block the detrimental functions of neutrophils and promote their beneficial actions. Therefore, further investigations are needed to increase our understanding of the molecular drivers of neutrophil development and plasticity to open new therapeutic avenues.

## Author contributions

SC, ID, GG, AR wrote the manuscript and prepare figures, these authors contributed equally to this work. EB and SJ revised the manuscript. All authors contributed to the article and approved the submitted version.
